# A state-wide education program on opioid use disorder: influential community members’ knowledge, beliefs, and opportunities for coalition development

**DOI:** 10.1186/s12889-022-13248-z

**Published:** 2022-05-04

**Authors:** Lindsey Hohmann, Haley Phillippe, Karen Marlowe, Ruth Jeminiwa, Natalie Hohmann, Salisa Westrick, Amanda Fowler, Brent Fox

**Affiliations:** 1 Department of Pharmacy Practice, Auburn University Harrison College of Pharmacy, 2316 Walker Building, Auburn, AL 36849 USA; 2 Department of Pharmacy Practice, Thomas Jefferson University College of Pharmacy, 901 Walnut Street, Health Professions Academic Building, Philadelphia, PA 19107 USA; 3 Department of Health Outcomes Research and Policy, Auburn University Harrison College of Pharmacy, 2316 Walker Building, Auburn, AL 36849 USA; 4 Division of Post Graduate Education, Auburn University Harrison College of Pharmacy, 2316 Walker Building, Auburn, AL 36849 USA

**Keywords:** Community coalition, Opioid use disorder, Interprofessional, Training program, Department of mental health

## Abstract

**Background:**

Deep South states, particularly Alabama, experience disproportionately higher opioid prescribing rates versus national rates. Considering limited opioid use disorder (OUD) providers in this region, collaborative efforts between non-healthcare professionals is critical in mitigating overdose mortality. The Alabama Opioid Training Institute (OTI) was created in 2019 to empower community members to take action in combatting OUD in local regions. The OTI included: 1) eight full-day in-person conferences; and 2) an interactive mobile-enabled website (https://alabamaoti.org). This study assessed the impact of the OTI on influential community members’ knowledge, abilities, concerns, readiness, and intended actions regarding OUD and opioid overdose mitigation.

**Methods:**

A one-group prospective cohort design was utilized. Alabama community leaders were purposively recruited via email, billboards, television, and social media advertisements. Outcome measures were assessed via online survey at baseline and post-conference, including: OUD knowledge (percent correct); abilities, concerns, and readiness regarding overdose management (7-point Likert-type scale, 1 = strongly disagree to 7 = strongly agree); and actions/intended actions over the past/next 6 months (8-item index from 0 to 100% of the time). Conference satisfaction was also assessed. Changes were analyzed using McNemar or Marginal Homogeneity tests for categorical variables and two-sided paired t-tests for continuous variables (alpha = 0.05).

**Results:**

Overall, 413 influential community members participated, most of whom were social workers (25.7%), female (86.4%), and White (65.7%). Community members’ OUD knowledge increased from mean [SD] 71.00% [13.32] pre-conference to 83.75% [9.91] post-conference (*p* < 0.001). Compared to pre-conference, mean [SD] ability scale scores increased (3.72 [1.55] to 5.15 [1.11], *p* < 0.001) and concerns decreased (3.19 [1.30] to 2.64 [1.17], *p* < 0.001) post-conference. Readiness was unchanged post-conference. Attendees’ intended OUD-mitigating actions in the next 6 months exceeded their self-reported actions in the past 6 months, and 92% recommended the OTI to others.

**Conclusions:**

The Alabama OTI improved community leaders’ knowledge, abilities, and concerns regarding OUD management. Similar programs combining live education and interactive web-based platforms can be replicated in other states.

**Supplementary Information:**

The online version contains supplementary material available at 10.1186/s12889-022-13248-z.

## Background

Opioid misuse continues to be a major public health issue in the United States. Over 150 million prescriptions for opioid pain relievers were written in 2019, and nearly 47,000 people die annually due to opioid overdose [1]. This is especially critical in the Deep South, as the opioid prescribing rate is disproportionately higher in this region, with the state of Alabama having the highest opioid prescribing rate in the nation [2, 3]. Furthermore, dispensing and overdose rates differ in counties within Alabama [1], with only 210 substance use disorder treatment facilities [4], 95 prescribers authorized to provide buprenorphine treatment for opioid use disorder (OUD) [5], and 104 naloxone access points across the state [6]. Limited resources and healthcare personnel necessitate a grass-roots approach and make collaborative efforts between influential community members, including community leaders and non-healthcare professionals, critical in mitigating opioid misuse and overdose mortality [7].

Despite the need for community member involvement, most lack training regarding opioid use disorder. In fact, 20% of the U.S. public reports limited knowledge of policies regarding OUD risk mitigation and 51% are unaware of effective treatment options for OUD [8]. Alabamians in particular express a lack of understanding of the potential progression from legitimate prescription opioid use to misuse of illicit opioids [9]. Furthermore, although multiple events have been conducted to train law enforcement officers and first responders on naloxone administration [10–12], limited work has been done to bring together community leaders and provide multifaceted training on the etiology of opioid misuse and potential treatment options and solutions [13]. Empowering the individuals that live and work in Alabama communities can lead to targeted efforts to mitigate OUD in the most needed areas, especially among hard-to-reach individuals that do not see healthcare providers regularly but may confide in local community leaders working in civil service, faith-based organizations, schools, social work, or the justice system [14–16]. This empowerment of influential community members is the first step in developing community coalitions that can augment healthcare professionals by providing an accessible, trusted source of knowledge and referral to local resources [15–17], ultimately improving the health outcomes and social welfare of people who use opioids in Alabama.

Accordingly, the Alabama Opioid Training Institute (OTI) was created using an interprofessional model to increase awareness and empower influential community members to take action in combatting OUD in their local regions. The purpose of this study was to assess the impact of the OTI program on community leaders’ knowledge, abilities, concerns, readiness, and intended actions regarding OUD and opioid overdose management in Alabama.

## Methods

### Study design

The Alabama OTI is a program implemented by the Auburn University Harrison College of Pharmacy and Alabama Department of Mental Health to empower influential community members and healthcare providers to take action in combatting OUD and assess the effect of multifaceted education on enhancing ability to intervene in cases of opioid misuse or overdose (http://pharmacy.auburn.edu/oti/). Separate conference series geared towards influential community members versus healthcare providers were developed; this paper describes the results of the community conferences series. A one-day conference was developed and delivered from May–August 2019 in seven cities across Alabama. Specifically, this study utilized a quasi-experimental one-group non-controlled prospective cohort design to assess change in influential community members’ knowledge, abilities, concerns, readiness, and intended actions regarding opioid misuse and overdose management before and after the conference. All study procedures were reviewed and the need for ethics approval was formally waived by the Institutional Review Board (IRB) at the primary author’s institution (Protocol # 21–188 EX 2104).

### Participants, setting, and recruitment

Influential community members, defined broadly as those who represent or have a prominent position in a community/organization, were purposively recruited to participate in the conference using a website hosted at the authors’ institution, email advertisements distributed to professional organizations and state boards, Facebook advertisements, digital billboards, and digital television advertisements in restaurants and businesses across Alabama. Adults ≥18 years-of-age living and/or working in the state of Alabama were eligible to participate. To maximize potential reach and impact in terms of preventing opioid misuse and overdose mortality, recruitment efforts were targeted towards individuals employed in the following professions: civil servants; mental health counselors; emergency medical technicians (EMTs); law enforcement; guidance counselors; school nurses; teachers (K-12); or faith-based organizations. Other non-healthcare professionals were welcome to attend, but healthcare providers were encouraged to attend the separate OTI conference series developed specifically to address treatment guidelines and best practices (described elsewhere).

### Opioid Training Institute (OTI) program components

Program components consisted of: 1) a collaborative educational conference; and 2) web-based resources.

#### Collaborative educational conference

An 8-h conference was developed by experts in chronic pain management and OUD and delivered in-person at no charge to participants 8 times at 7 distinct locations across Alabama. Expert speakers were recruited from medicine, pharmacy, law enforcement, and local community organizations to increase the conference’s legitimacy and relevancy. Educational programming consisted of 5 topics: 1) overview of the U.S. and Alabama opioid crisis (1 h); 2) understanding and recognizing OUD (2 h); 3) Medication Assisted Treatment (MAT) (0.67 h); 4) naloxone administration (0.5 h); and 5) overview of treatment solutions and resources (1 h). The conference concluded with a panel discussion (0.67 h). The schedule also included breaks and lunch, during which there was no programming.

#### Web-based resources

To support sustained community-based efforts to mitigate opioid misuse and overdose mortality, a mobile-enabled online platform was developed that incorporated: 1) educational materials and webinars; 2) links to local community resources for OUD treatment and naloxone access; and 3) interactive “test-your-knowledge” quiz questions (https://alabamaoti.org). Educational topics included opioid crisis statistics, naloxone instructions and training video, and OUD treatment options, with webinars focusing on culturally competent communication for opioid misuse prevention conversations and successful community programs.

### Data collection and measures

Program impact was assessed via online survey distributed by email at baseline (pre) and post-conference. Primary outcome measures included: knowledge regarding OUD and overdose; abilities, concerns, and readiness regarding managing an opioid overdose; and self-reported actions/intended actions over the past/next 6 months regarding OUD and overdose mitigation. Satisfaction with the conference was also assessed. Knowledge was measured using a 13-item multiple-choice scale adapted from the Opioid Overdose Knowledge Scale (OOKS) described by Williams et al. [18]. Perceived ability to manage an opioid overdose (10-items), concerns regarding managing an opioid overdose (8-items), and readiness to manage an opioid overdose (10-items) were measured using 7-point Likert-type scales from 1 = strongly disagree to 7 = strongly agree adapted from the Opioid Overdose Attitude Scale (OOAS) described by Williams et al. [18] and Nielsen et al. [19]. Self-reported behavior over the past 6 months (pre-conference) and intended behavior over the next 6 months (post-conference) were measured using an 8-item index informed by Lynn et al. [20] and Nielsen et al. [19]. Attendees were asked to rate the percent of time they took action/intended to take action when presented with a situation related to opioid misuse or overdose in the past/next 6 months (0–20%, 21–40%, 41–60%, 61–80%, or 81–100% of the time). Satisfaction with the level of training received through the conference (9-items) as well as general satisfaction with the conference’s content and format (9-items) were measured using 7-point Likert-type scales from 1 = strongly disagree to 7 = strongly agree. The survey developed for this study is provided as Additional file 1 (baseline survey) and Additional file 2 (post-conference survey); no survey instruments in this study are under license.

### Data analysis

Demographic, primary outcome, and satisfaction data were characterized using descriptive statistics (frequency, percentage, mean, standard deviation). Changes in the proportion of attendees who correctly answered knowledge items, the proportion of self-reported actions/intentions, or the frequency of agreement with perceived ability, concerns, and readiness items from pre- to post-conference were analyzed using McNemar’s or Marginal Homogeneity tests as appropriate. Changes in mean knowledge score (percent of questions answered correctly) and mean ability, concerns, and readiness scale scores from pre to post were evaluated using two-sided paired t-tests. Internal consistency of scale constructs was measured using KR-20 (knowledge) or Cronbach’s alpha (ability, concerns, readiness). Exploratory factor analysis was performed using principle components analysis and direct oblimin rotation to assess validity of perceived ability, concerns, and readiness constructs. Components with eigenvalues > 1.5 were retained and scale items with factor loadings < 0.600 were dropped from analysis. Analyses were conducted using SPSS Statistical Software version 24 (IBM, Armonk, New York) with alpha = 0.05.

## Results

### Conference attendance and baseline characteristics

The community conference was held eight times at seven locations in 2019 with a total of 413 participants (Table 1). There was a wide representation of different professions at the conference. The highest attendance was from social workers (25.7%), with mental health counselors, faith-based organizations, local business owners, civil servants, law enforcement, emergency medical technicians, school nurses, and school teachers (K-12) also in attendance. The majority of attendees were female (86.4%) and White (65.7%) with a mean age of 45 years.

**Table 1:** Community conference attendee characteristics at baseline (*N* = 413)^a^

Question	***n*** (%)
Profession	
Civic official or city servant	4 (1.0)
Emergency Medical Technician (EMT)	3 (0.70)
Guidance counselor	4 (1.0)
Lawyer	2 (0.50)
Mental health counselor	43 (10.5)
School nurse	16 (3.9)
Social worker	105 (25.7)
Behavioral health specialist	10 (2.2)
Community member	4 (1.0)
Faith-based organization or church official	15 (3.7)
Law enforcement	14 (3.4)
Medication Assisted Treatment (MAT) provider	7 (1.7)
School teacher (K-12)	9 (2.2)
Sex	
Male	56 (13.6)
Female	355 (86.4)
Race	
White/Caucasian	266 (65.7)
Black/African American	126 (31.1)
Asian or Pacific Islander	3 (0.70)
Native American or Alaska Native	1 (0.20)
Other	9 (2.2)
Ethnicity	
Hispanic Origin	16 (4.0)
Non-Hispanic Origin	387 (96.0)
Participated in other opioid-related education/training in past 6 months	
No	319 (77.4)
Yes	93 (22.6)
Do you know or have you ever known anyone in your personal or professional life who has struggled with opioid use disorder?	
No	104 (25.4)
Yes	305 (74.6)
Offer services or programs related to opioid use disorder	
No	207 (51.6)
Yes	194 (48.4)
Methadone program/provision	25 (6.2)
Buprenorphine or buprenorphine/naloxone provision	26 (6.5)
Needle exchange program	1 (0.2)
Cognitive behavioral therapy or counseling	91 (22.7)
Medication disposal or drug take-back	22 (5.5)
Education sessions or programs	94 (23.4)
Other	37 (9.2)
	**Mean (SD)**
Age, years	45.3 (12.6)

^a^Percentages may differ due to item non-response

### Knowledge

Internal consistency of the knowledge measure was moderate (KR-20 = 0.538). Overall, community leaders answered more questions correctly after attending the conference compared to before the conference (mean [SD]: 83.75% [9.91] post vs. 71.00% [13.32] pre, *p* < 0.001) (Table 2). Specifically, there was a statistically significant improvement in the number of participants answering correctly on 10 of 13 items from pre- to post-conference. Of note, over 90% of respondents correctly answered 3 of the 13 items at baseline, with no statistically significant improvement on these questions after the conference (*p* = 0.180, *p* = 1.000, and *p* = 0.855).

**Table 2:** Community attendees’ knowledge pre- and post-conference (*N* = 300)^a^

		**Mean (SD)**		
**Measure**	**KR-20**	**Pre**	**Post**	***p*-value** ^**b**^
Percent of Knowledge Questions Answered Correctly	0.538	71.00 (13.32)	83.75 (9.91)	< 0.001*
**Question**		**Frequency of Correct Response, n (%)**^**c**^		
		**Pre**	**Post**	***p*-value** ^**d**^
Fentanyl is the number one drug leading to opioid overdose deaths nationwide				
*Correct response: True*		248 (84.9)	276 (92.3)	0.002*
Multiple doses of naloxone may not be effective in reversing overdose from the following opioid				
*Correct response: Carfentanil*		116 (42.8)	234 (78.3)	< 0.001*
Which of the following mental and social factors are shown to influence risk for opioid misuse, especially in adolescents?				
*Correct response: All of the following* *Level of self-esteem; Resiliency (coping and problem-solving skills); Stress of feelings of inadequacy; Behavioral disorders; and Bullying*		284 (96.9)	294 (98.0)	0.180
Over time, opioid use disorder affects individuals’ ability to				
*Correct response: All of the following* *Regulate behavior; Make decisions; and Respond to stressful situations*		291 (99.3)	297 (99.3)	1.000
Which of the following are indicators of an opioid overdose?^e^				
*Correct response: All of the following*		8 (2.8)	46 (15.4)	< 0.001*
*Slow or shallow breathing*		*246 (82.0)*	*294 (98.0)*	
*Lips, hands or feet turning blue*		*193 (64.3)*	*277 (92.3)*	
*Loss of consciousness*		*254 (84.7)*	*282 (94.0)*	
*Unresponsive*		*262 (87.3)*	*288 (96.0)*	
*Deep snoring*		*88 (29.3)*	*204 (68.0)*	
*Very small pupils*		*126 (42.0*)	*188 (62.7)*	
*Incorrect responses:*		277 (97.2)	253 (84.6)	
*Having blood-shot eyes*		*60 (20.0)*	*69 (23.0)*	
*Seizing*		*170 (56.7)*	*141 (47.0)*	
*Agitated behavior*		*103 (34.3)*	*107 (35.7)*	
*Rapid heartbeat*		*115 (38.3)*	*98 (32.7)*	
Which of the following should be done when managing a heroin / opioid overdose? ^e^				
*Correct response: All of the following*		124 (42.0)	222 (74.0)	< 0.001*
*Call an ambulance (911)*		*292 (97.3)*	*297 (99.0)*	
*Give naloxone (opioid overdose antidote)*		*216 (72.0)*	*289 (96.3)*	
*Stay with the person until help arrives*		*257 (85.7)*	*292 (97.3)*	
*Check for responsiveness*		*212 (70.7)*	*253 (84.3)*	
*Give chest compressions and/or rescue breathing*		*201 (67.0)*	*267 (89.0)*	
*Incorrect responses:*		171 (58.0)	78 (26.0)	
*Inject the person with salt solution or milk*		*2 (0.70)*	*2 (0.70)*	
*Give stimulants (*e.g. *cocaine or black coffee)*		*7 (2.3)*	*2 (0.70)*	
*Put the person in a bath of cold water*		*10 (3.3)*	*7 (2.3)*	
*Put the person in bed to sleep it off*		*3 (1.0)*	*1 (0.30)*	
What is naloxone used for?				
*Correct response: To reverse the effects of an opioid overdose (*e.g. *heroin, methadone)*		249 (86.8)	283 (95.0)	0.001*
How long does naloxone take to have an effect?				
*Correct response: Within 5 min*		253 (89.1)	280 (93.6)	0.049*
How long do the effects of naloxone last for?				
*Correct response: 30–90 min*		155 (58.5)	211 (71.5)	< 0.001*
Which of the following is NOT used in medication assisted treatment (MAT) to treat opioid use disorder?				
*Correct response: Hydromorphone*		170 (65.4)	229 (77.6)	< 0.001*
Methadone is the treatment of choice for pregnant women with opioid use disorder				
*Correct response: True*		167 (63.0)	251 (84.8)	< 0.001*
Which of the following is a 12-step program developed to help individuals with substance use disorder?				
*Correct response: Narcotics Anonymous*		257 (90.5)	270 (90.6)	0.855
Some individuals may use more opioids in an attempt to relieve depression that occurs with their chronic pain				
*Correct response: True*		275 (96.5)	297 (99.3)	0.021*

^a^Attendees are matched across time points (Pre *n* = 300, Post *n* = 300)

^b^Results of paired-sample t-test. Significance at the alpha = 0.05 level indicated by*

^c^Percentages may differ due to item non-response

^d^Results of McNemar test. Significance at the alpha = 0.05 level indicated by*

^e^Respondents were asked to check all that applied

### Perceptions regarding managing an opioid overdose: abilities, concerns, and readiness

Exploratory factor analysis showed that scale items loaded on 3 factors with eigenvalues > 1.5 consistent with: 1) perceived ability to manage an opioid overdose; 2) concerns regarding managing an opioid overdose; and 3) readiness to intervene in an opioid overdose situation. Internal consistency of ability (Cronbach’s alpha = 0.919), concerns (0.842), and readiness (0.840) scales was high (Tables 3, 4 and 5).

**Table 3:** Community attendees’ ability to manage an opioid overdose pre- and post-conference (*N* = 300)^a^

**Measure**	**Cronbach’s Alpha**		**Mean (SD)**^**b**^						
			**Pre**			**Post**			***p*-value**^**c**^
Ability Scale Score	0.919		3.72 (1.55)			5.16 (1.11)			< 0.001*
**Question**		**n (%)**^**d**^							
	**Time**	**1**	**2**	**3**	**4**	**5**	**6**	**7**	***p*-value**^**e**^
I already have enough information about how to manage an overdose	**Pre**	**126 (43.6)**	74 (25.6)	24 (8.3)	25 (8.7)	24 (8.3)	9 (3.1)	7 (2.4)	< 0.001*
	**Post**	12 (4.1)	35 (11.9)	26 (8.8)	56 (19.0)	68 (23.1)	**74 (25.1)**	24 (8.1)	
I am already able to administer naloxone to someone who has overdosed	**Pre**	**153 (53.3)**	51 (17.8)	10 (3.5)	17 (5.9)	15 (5.2)	22 (7.7)	19 (6.6)	< 0.001*
	**Post**	26 (8.8)	37 (12.6)	21 (7.1)	39 (13.3)	49 (16.7)	**86 (29.3)**	36 (12.2)	
I would be able to check that someone who has overdosed was breathing properly	**Pre**	**58 (20.2)**	28 (9.8)	21 (7.3)	23 (8.0)	53 (18.5)	51 (17.8)	53 (18.5)	< 0.001*
	**Post**	3 (1.0)	12 (4.1)	6 (2.0)	32 (10.9)	50 (17.0)	**121 (41.2)**	70 (23.8)	
I am going to need more training before I would feel confident to help someone who has overdosed^f,g^	**Pre**	32 (11.1)	17 (5.9)	16 (5.6)	25 (8.7)	38 (13.2)	68 (23.6)	**92 (31.9)**	< 0.001*
	**Post**	25 (8.5)	**63 (21.4)**	41 (12.9)	43 (14.6)	51 (17.3)	46 (15.6)	26 (8.8)	
I would be able to perform mouth-to-mouth resuscitation on someone who has overdosed	**Pre**	40 (13.9)	26 (9.0)	16 (5.6)	23 (8.0)	59 (20.5)	61 (21.2)	**63 (21.9)**	< 0.001*
	**Post**	7 (2.4)	18 (6.1)	22 (7.5)	41 (13.9)	43 (14.6)	**104 (35.3)**	60 (20.3)	
I would be able to perform chest compressions on someone who has overdosed	**Pre**	33 (11.5)	19 (6.6)	11 (3.8)	22 (7.6)	60 (20.8)	**75 (26.0)**	68 (23.6)	< 0.001*
	**Post**	5 (1.7)	11 (3.7)	17 (5.8)	23 (7.8)	48 (16.3)	**113 (38.3)**	78 (26.4)	
If someone overdoses, I would know what to do to help them	**Pre**	39 (13.6)	46 (16.0)	38 (13.2)	34 (11.8)	**66 (23.0)**	38 (13.2)	26 (9.1)	< 0.001*
	**Post**	–	4 (1.4)	9 (3.1)	23 (7.8)	67 (22.7)	**124 (42.0)**	68 (23.1)	
I would be able to place someone who has overdosed in the recovery position	**Pre**	**52 (18.1)**	**52 (18.1)**	35 (12.2)	28 (9.7)	42 (14.6)	38 (13.2)	41 (14.2)	< 0.001*
	**Post**	4 (1.4)	10 (3.4)	17 (5.8)	43 (14.7)	52 (17.7)	**104 (35.5)**	63 (21.5)	
I know very little about how to help someone who has overdosed^f,g^	**Pre**	32 (11.3)	**54 (19.0)**	33 (11.6)	42 (14.8)	42 (14.8)	39 (13.7)	42 (14.8)	< 0.001*
	**Post**	59 (20.2)	**107 (36.6)**	52 (17.8)	39 (13.4)	15 (5.1)	18 (6.2)	2 (0.70)	
I would be able to deal effectively with an overdose	**Pre**	**59 (20.5)**	42 (14.6)	29 (10.1)	57 (19.8)	49 (17.0)	34 (11.8)	18 (6.3)	< 0.001*
	**Post**	5 (1.7)	12 (4.1)	21 (7.1)	54 (18.3)	72 (24.4)	**93 (31.5)**	38 (12.9)	

1 = strongly disagree, 2 = disagree, 3 = somewhat disagree, 4 = neutral, 5 = somewhat agree, 6 = agree, 7 = strongly agree

^a^Attendees are matched across time points (Pre *n* = 300, Post *n* = 300)

^b^On a scale of 1 to 7 where 1 = strongly disagree and 7 = strongly agree

^c^Results of paired-sample t-test. Significance at the alpha = 0.05 level indicated by*

^d^Percentages may differ due to item non-response

^e^Results of Marginal Homogeneity test. Significance at the alpha = 0.05 level indicated by*

^f^Survey items were reverse coded when assessing mean scale scores

^g^Factor loading < 0.600. Excluded from analysis of mean scale scores

**Table 4:** Community attendees’ concerns regarding managing an opioid overdose pre- and post-conference (*N* = 300)^a^

		**Mean (SD)**^**b**^							
**Measure**	**Cronbach’s Alpha**	**Pre**			**Post**				***p*-value**^**c**^
Concerns Scale Score	0.842	3.19 (1.30)			2.64 (1.17)				< 0.001*
**Question**		**n (%)**^**d**^							
	**Time**	**1**	**2**	**3**	**4**	**5**	**6**	**7**	***p*-value**^**e**^
I would be afraid of giving naloxone in case the person becomes aggressive afterwards	**Pre**	44 (15.3)	**83 (28.9)**	26 (9.1)	54 (18.8)	35 (12.2)	26 (9.1)	19 (6.6)	< 0.001*
	**Post**	75 (25.6)	**108 (36.9)**	39 (13.3)	36 (12.3)	21 (7.2)	6 (2.0)	8 (2.7)	
I would be afraid of doing something wrong in an overdose situation	**Pre**	25 (8.7)	64 (22.3)	18 (6.3)	38 (13.2)	**66 (23.0)**	46 (16.0)	30 (10.5)	< 0.001*
	**Post**	48 (16.6)	**77 (26.6)**	35 (12.1)	47 (16.2)	56 (19.3)	17 (5.9)	10 (3.4)	
I would be reluctant to use naloxone for fear of precipitating withdrawal symptoms	**Pre**	48 (16.7)	**85 (29.6)**	39 (13.6)	54 (18.8	24 (8.4)	23 (8.0)	14 (4.9)	< 0.001*
	**Post**	85 (29.1)	**109 (37.3)**	43 (14.7)	31 (10.6)	12 (4.1)	9 (3.1)	3 (1.0)	
I would be concerned about calling emergency services in case the police show up^g^	**Pre**	**177 (61.7)**	79 (27.5)	8 (2.8)	13 (4.5)	3 (1.0)	3 (1.0)	4 (1.4)	0.925
	**Post**	**168 (57.5)**	102 (34.9)	3 (1.0)	9 (3.1)	3 (1.0)	4 (1.4)	3 (1.0)	
If I tried to help someone who has overdosed, I might accidentally hurt them	**Pre**	50 (17.4)	**98 (34.1)**	33 (11.5)	55 (19.2)	29 (10.1)	11 (3.8)	11 (3.8)	< 0.001*
	**Post**	70 (23.9)	**115 (39.2)**	43 (14.7)	37 (12.6)	17 (5.8)	10 (3.4)	1 (0.30)	
I would feel safer if I knew that naloxone was around^f,g^	**Pre**	10 (3.5)	12 (4.2)	9 (3.2)	**67 (23.5)**	50 (17.5)	78 (27.4)	59 (20.7)	< 0.001*
	**Post**	3 (1.0)	5 (1.7)	6 (2.0)	48 (16.3)	45 (15.3)	**117 (39.8)**	70 (23.8)	
I would be afraid of suffering a needle stick injury if I had to give someone a naloxone injection	**Pre**	64 (22.3)	**82 (28.6)**	18 (6.3)	52 (18.1)	33 (11.5)	23 (8.0)	15 (5.2)	0.009*
	**Post**	56 (19.2)	**108 (37.1)**	32 (11.0)	41 (14.1)	29 (10.0)	17 (5.8)	8 (2.7)	
Needles frighten me, and I wouldn’t be able to give someone an injection of naloxone	**Pre**	**112 (39.0)**	76 (26.5)	22 (7.7)	39 (13.6)	24 (8.4)	8 (2.8)	6 (2.1)	0.057
	**Post**	**107 (36.8)**	101 (34.7)	25 (8.6)	30 (10.3)	18 (6.2)	6 (2.1)	4 (1.4)	

1 = strongly disagree, 2 = disagree, 3 = somewhat disagree, 4 = neutral, 5 = somewhat agree, 6 = agree, 7 = strongly agree

^a^Attendees are matched across time points (Pre *n* = 300, Post *n* = 300)

^b^On a scale of 1 to 7 where 1 = strongly disagree and 7 = strongly agree

^c^Results of paired-sample t-test. Significance at the alpha = 0.05 level indicated by*

^d^Percentages may differ due to item non-response

^e^Results of Marginal Homogeneity test. Significance at the alpha = 0.05 level indicated by*

^f^Survey items were reverse coded when assessing mean scale scores

^g^Factor loading < 0.600. Excluded from analysis of mean scale scores

**Table 5:** Community attendees’ readiness to manage an opioid overdose pre- and post-conference (*N* = 300)^a^

			**Mean (SD)**^**b**^						
**Measure**	**Cronbach’s Alpha**		**Pre**			**Post**			***p*-value**^**c**^
Readiness Scale Score	0.840		6.51 (0.70)			6.47 (0.66)			0.384
**Question**		**n (%)**^**d**^							
	**Time**	**1**	**2**	**3**	**4**	**5**	**6**	**7**	***p*-value**^**e**^
Everyone at risk of witnessing an overdose should have naloxone^g^	**Pre**	10 (3.5)	27 (9.5)	10 (3.5)	69 (24.2)	39 (13.7)	61 (21.4)	**69 (24.2)**	< 0.001*
	**Post**	7 (2.4)	2 (0.70)	1 (0.30)	26 (8.9)	37 (12.7)	**113 (38.7)**	106 (36.3)	
I couldn’t just watch someone overdose, I would have to do something to help	**Pre**	1 (0.30)	3 (1.0)	2 (0.70)	10 (3.5)	22 (7.6)	89 (30.9)	**161 (55.9)**	0.374
	**Post**	1 (0.30)	–	2 (0.70)	8 (2.7)	17 (5.8)	108 (36.9)	**157 (53.6)**	
If someone overdoses, I would call an ambulance, but I wouldn’t be willing to do anything else^f,g^	**Pre**	**98 (34.0)**	93 (32.3)	42 (14.6)	23 (8.0)	11 (3.8)	8 (2.8)	13 (4.5)	0.170
	**Post**	**109 (37.3)**	96 (32.9)	39 (13.4)	20 (6.8)	9 (3.1)	9 (3.1)	10 (3.4)	
Family and friends of drug users should be prepared to deal with an overdose^g^	**Pre**	6 (2.1)	6 (2.1)	3 (1.0)	16 (5.6)	17 (5.9)	82 (28.6)	**157 (54.7)**	0.008*
	**Post**	4 (1.4)	–	2 (0.70)	9 (3.1)	17 (5.8)	91 (31.2)	**169 (57.9)**	
If I saw an overdose, I would panic and not be able to help^f,g^	**Pre**	**95 (32.9)**	95 (32.9)	34 (11.8)	33 (11.4)	10 (3.5)	7 (2.4)	15 (5.2)	< 0.001*
	**Post**	**115 (39.4)**	**115 (39.4)**	33 (11.3)	13 (4.5)	10 (3.4)	3 (1.0)	3 (1.0)	
If I witnessed an overdose, I would call an ambulance immediately	**Pre**	4 (1.4)	2 (0.70)	1 (0.30)	6 (2.1)	5 (1.7)	49 (17.0)	**221 (76.7)**	0.566
	**Post**	1 (0.30)	1 (0.30)	–	6 (2.0)	11 (3.8)	76 (25.9)	**198 (67.6)**	
I would stay with the overdose victim until help arrives	**Pre**	2 (0.70)	–	–	6 (2.1)	8 (2.8)	52 (18.1)	**219 (76.3)**	0.211
	**Post**	–	–	–	7 (2.4)	7 (2.4)	83 (28.2)	**197 (67.0)**	
If I saw an overdose, I would feel nervous, but I would still take the necessary actions ^g^	**Pre**	5 (1.7)	5 (1.7)	6 (2.1)	15 (5.2)	24 (8.3)	106 (36.7)	**128 (44.3)**	0.547
	**Post**	6 (2.0)	4 (1.4)	3 (1.0)	18 (6.1)	29 (9.9)	**119 (40.5)**	115 (39.1)	
I will do whatever is necessary to save someone’s life in an overdose situation	**Pre**	2 (0.70)	2 (0.70)	–	12 (4.2)	23 (8.0)	83 (28.8)	**166 (57.6)**	0.587
	**Post**	1 (0.30)	–	–	16 (5.5)	19 (6.5)	110 (37.5)	**147 (50.2)**	
If someone overdoses, I want to be able to help them	**Pre**	2 (0.70)	–	1 (0.30)	7 (2.4)	6 (2.1)	65 (22.6)	**207 (71.9)**	0.068
	**Post**	2 (0.70)	–	–	9 (3.1)	10 (3.4)	92 (31.3)	**181 (61.6)**	

1 = strongly disagree, 2 = disagree, 3 = somewhat disagree, 4 = neutral, 5 = somewhat agree, 6 = agree, 7 = strongly agree

^a^Attendees are matched across time points (Pre *n* = 300, Post *n* = 300)

^b^On a scale of 1 to 7 where 1 = strongly disagree and 7 = strongly agree

^c^Results of paired-sample t-test. Significance at the alpha = 0.05 level indicated by*

^d^Percentages may differ due to item non-response

^e^Results of Marginal Homogeneity test. Significance at the alpha = 0.05 level indicated by*

^f^Survey items were reverse coded when assessing mean scale scores

^g^Factor loading < 0.600. Excluded from analysis of mean scale scores

#### Perceived ability to manage an opioid overdose

Overall, there was a statistically significant increase in perceived ability to manage an opioid overdose from pre- to post-conference (Table 3), with an increase in the ability scale score from mean (SD) 3.72 (1.55) to 5.15 (1.11) (*p* < 0.001). Responses to all scale items followed this same positive trend from baseline to post-conference, including knowing what to do in an opioid overdose situation (22.3 to 65.1% agreed/strongly agreed; *p* < 0.001), administering naloxone (14.3 to 41.5%; *p* < 0.001), and placing someone in the recovery position (27.4 to 57.0%; *p* < 0.001).

#### Concerns regarding managing an opioid overdose

Similarly, there was a statistically significant decrease in overall concerns regarding managing an opioid overdose from pre- to post-conference (Table 4), with a decrease from mean (SD) 3.19 (1.30) to 2.64 (1.17) (*p* < 0.001). Of note, 6 of the 8 scale items followed this trend of decreasing concerns from baseline to post-conference, including concerns regarding aggression after naloxone administration (15.7 to 4.7% agreed/strongly agreed; *p* < 0.001) and doing something wrong in an opioid overdose situation (26.5 to 9.3%; *p* < 0.001). However, there was no change in level of agreement regarding concerns about fear of needles (4.9 to 3.5%; *p* = 0.057) or police showing up after calling emergency services (2.4 to 2.4%, *p* = 0.925).

#### Readiness to intervene in an opioid overdose situation

There was no statistically significant change in overall readiness to manage an opioid overdose from pre (mean [SD] 6.51 [0.70]) to post (6.47 [0.66]; *p* = 0.384) (Table 5). In general, there was a high level of readiness/willingness to manage on opioid overdose at baseline, with no change in level of readiness on 7 of 10 scale items. Three items differed in the level of readiness from pre- to post-conference, including the belief that: everyone at risk of witnessing an overdose should have naloxone (45.6 to 75.0% agreed/strongly agreed; *p* < 0.001); family and friends of drug users should be prepared to deal with an overdose (83.3 to 89.1%; *p* = 0.008); and the respondent would panic and not be able to help in an overdose situation (7.6 to 2.0%; *p* < 0.001).

### Actions and intended actions

In general, attendees’ intended actions related to opioid misuse or overdose management in the next 6 months exceeded their self-reported actions in the past 6 months (Table 6). In the past 6 months, 5.2% of attendees educated family or caregivers about OUD between 81 and 100% of the time when presented with the opportunity; after the conference, 13.7% of attendees intended to provide this education at least 81% of the time (*p* < 0.001). Additionally, 3.5% of participants recommended or discussed naloxone at least 81% of the time when presented with the opportunity in the past 6 months, whereas 15.8% intended to do so in the next 6 months (*p* < 0.001).

**Table 6:** Community attendees’ actions in the past 6 months (pre-conference) and intended actions in the next 6 months (post-conference) (*N* = 300)

Action	Time^**b**^	Percent of Time Actions Taken When Presented with the Opportunity n (%)^**a**^						***p***-value^**d**^
		0–20%	21–40%	41–60%	61–80%	81–100%	N/A^**c**^	
Screen or assess someone for potential opioid use disorder (OUD) or opioid overdose risk	**Pre**	**83 (29.1)**	16 (5.6)	14 (4.9)	12 (4.2)	22 (7.7)	138 (48.4)	< 0.001*
	**Post**	**93 (31.5)**	39 (13.2)	17 (5.8)	17 (5.8)	38 (12.9)	91 (30.8)	
Educate people about OUD through school or community-based programs	**Pre**	**86 (30.0)**	12 (4.2)	17 (5.9)	9 (3.0)	24 (8.4)	139 (48.4)	< 0.001*
	**Post**	**98 (33.4)**	48 (16.4)	31 (10.6)	18 (6.1)	42 (14.3)	56 (19.1)	
Provide education or counseling to family or caregivers regarding OUD	**Pre**	**86 (30.1)**	21 (7.3)	20 (7.0)	9 (3.1)	15 (5.2)	135 (47.2)	< 0.001*
	**Post**	**102 (35.1)**	46 (15.8)	28 (9.6)	20 (6.9)	40 (13.7)	55 (18.9)	
Recommend or discuss specialized treatment or rehabilitation facilities for a person with OUD	**Pre**	**93 (32.4)**	21 (7.3)	23 (8.0)	15 (5.2)	19 (6.6)	116 (40.4)	< 0.001*
	**Post**	**98 (33.6)**	33 (11.3)	26 (8.9)	23 (7.9)	47 (16.1)	65 (22.3)	
Recommend or discuss cognitive behavioral therapy for OUD	**Pre**	**79 (27.5)**	28 (9.8)	16 (5.6)	10 (3.5)	16 (5.6)	138 (48.1)	< 0.001*
	**Post**	**98 (33.6)**	34 (11.6)	28 (9.6)	21 (7.2)	37 (12.7)	74 (25.3)	
Recommend or discuss medication assisted treatment for OUD	**Pre**	**87 (30.5)**	19 (6.7)	17 (6.0)	3 (1.1)	18 (6.3)	141 (49.5)	< 0.001*
	**Post**	**99 (33.8)**	32 (10.9)	29 (9.9)	15 (5.1)	36 (12.3)	82 (28.0)	
Recommend or discuss naloxone	**Pre**	**91 (31.8)**	21 (7.3)	16 (5.6)	4 (1.4)	10 (3.5)	144 (50.3)	< 0.001*
	**Post**	**100 (34.4)**	36 (12.4)	21 (7.2)	24 (8.2)	46 (15.8)	64 (22.0)	
Speak with a healthcare provider on someone’s behalf	**Pre**	**93 (32.4)**	23 (8.0)	24 (8.4)	8 (2.8)	17 (5.9)	122 (42.5)	< 0.001*
	**Post**	**96 (33.0)**	28 (9.6)	36 (12.4)	24 (8.2)	37 (12.7)	70 (24.1)	

^a^Percentages may differ due to item non-response

^b^Attendees are matched across time points (Pre *n* = 300, Post *n* = 300)

^c^*N/A* no opportunities or not applicable

^d^Results of Marginal Homogeneity test. “N/A” answer choice was excluded from analysis. Significance at the alpha = 0.05 level indicated by*

### Satisfaction

Overall, attendees agreed or strongly agreed with the majority of items regarding satisfaction with the level of OUD management training and with the programming in general (Table 7). Over 66% agreed or strongly agreed that the training content was relevant to their job, 83.0% that the training increased their ability to recommend resources to individuals with OUD, and 76.6% that the training increased their ability to collaborate with others to prevent OUD. Furthermore, 90.1% agreed or strongly agreed that they were satisfied with the material presented during the program, and 91.7% would recommend the program to others.

**Table 7:** Community attendees’ satisfaction after the conference (*N* = 337)

Question	n (%) ^**a**^						
	1	2	3	4	5	6	7
**OUD and Overdose Management Training**							
All learning objectives for this educational program were met	2 (0.60)	–	2 (0.60)	6 (1.9)	24 (7.5)	**149 (46.7)**	136 (42.6)
Content was relevant to my job	2 (0.60)	2 (0.60)	8 (2.5)	45 (14.2)	49 (15.5)	**120 (38.0)**	90 (28.5)
The training materials were easy to read	–	–	–	17 (5.3)	19 (6.0)	**152 (47.8)**	130 (40.9)
The training adequately described strategies to prevent opioid use disorder (OUD)	2 (0.60)	1 (0.30)	2 (0.60)	19 (6.0)	29 (9.2)	**149 (47.2)**	114 (36.1)
The training adequately described strategies to treat OUD	–	–	1 (0.30)	10 (3.1)	23 (7.2)	**150 (47.2)**	134 (42.1)
The training adequately described strategies to communicate with individuals with OUD	–	1 (0.30)	2 (0.60)	14 (4.4)	32 (10.1)	**157 (49.5)**	111 (35.0)
After the training, my ability to recommend resources to individuals with OUD increased	1 (0.30)	1 (0.30)	–	19 (6.0)	33 (10.4)	**157 (49.4)**	107 (33.6)
After the training, my ability to recommend treatment to individuals with OUD increased	1 (0.30)	2 (0.60)	1 (0.30)	23 (7.3)	41 (13.0)	**151 (47.8)**	97 (30.7)
After the training, my ability to collaborate with others to prevent OUD increased	1 (0.30)	1 (0.30)	2 (0.60)	26 (8.2)	44 (13.9)	**146 (46.2)**	96 (30.4)
**General Program**							
The training content was clear and concise	–	–	3 (1.0)	5 (1.6)	22 (7.0)	**161 (51.1)**	124 (39.4)
Realistic time was allowed for the training	–	–	2 (0.60)	7 (2.2)	26 (8.3)	**162 (51.6)**	117 (37.3)
I was satisfied with the material presented during the program	–	–	2 (0.60)	9 (2.9)	20 (6.4)	**151 (48.2)**	131 (41.9)
I would recommend this program to others	–	–	2 (0.60)	7 (2.2)	17 (5.4)	131 (41.7)	**157 (50.0)**
The training met my educational needs	–	1 (0.30)	3 (1.0)	8 (2.6)	22 (7.1)	**150 (48.2)**	127 (40.8)
The quality of the facility was excellent	1 (0.30)	–	1 (0.30)	12 (3.8)	17 (5.4)	**141 (45.0)**	**141 (45.0)**
I have been pleased with the communication regarding the program	–	2 (0.60)	1 (0.30)	13 (4.2)	14 (4.5)	**145 (46.3)**	138 (44.1)
I have been pleased with the registration process for the program	–	1 (0.30)	2 (0.60)	8 (2.6)	18 (5.8)	132 (42.3)	**151 (48.4)**
The presenters were engaging	–	–	2 (0.60)	8 (2.6)	29 (9.4)	126 (40.8)	**144 (46.6)**

1 = strongly disagree, 2 = disagree, 3 = somewhat disagree, 4 = neutral, 5 = somewhat agree, 6 = agree, 7 = strongly agree

^a^Percentages may differ due to item non-response

## Discussion

The Alabama OTI was attended by a variety of community leaders and non-healthcare professionals, including those involved with faith-based organizations, civil service, the justice system, and K-12 schools with the potential to reach a large audience of in-need individuals. Although participants were employed in non-healthcare professions, most stated that the OTI was relevant to their job, suggesting that OUD mitigation is seen as a community-level issue versus solely the purview of healthcare providers. Additionally, although the goal of the OTI was not to create community coalitions for mitigating OUD, this study’s findings show that the foundational skills and knowledge needed for coalition formation were achieved and developed. Specifically, attendees’ knowledge and self-reported ability to manage an opioid overdose increased as a result of the program, with a decrease in concerns regarding how to handle an overdose situation.

Attendees reported increased knowledge regarding opioid drugs of concern, signs and symptoms of opioid overdose, medications to treat OUD, and naloxone administration. This is consistent with increases in knowledge seen after educational interventions among law enforcement officers [11], family members of people who use opioids [21], and general practitioners outside of Alabama [22]. Of note, there was no improvement in knowledge about social and mental risk factors for opioid misuse, the social consequences of opioid use disorder, or that Narcotics Anonymous is a 12-step program to help individuals with substance use disorder. However, over 90% of participants correctly answered questions related to these topics at baseline, making it difficult to detect a statistically significant increase and suggesting that influential community members were sufficiently informed on these topics prior to the conference. The presence of community-based educators, including community health workers (CHWs), in some regions may account for higher baseline knowledge on certain items in this study [23]. Future studies may partner with CHWs to elucidate information needs, sources, and dissemination modalities among leaders and non-healthcare professionals in particular communities, tailoring subsequent educational efforts to align with relevant and culturally appropriate information channels and efficiently utilize public health resources by targeting the most critical gaps in knowledge [23].

Regarding perceived ability to manage an opioid overdose, an overall improvement was seen from pre- to post-conference. In fact, statistically significant increases occurred for all items in the abilities scale. For example, after the conference, program participants more frequently reported that they would know what to do in an overdose situation in general, would be able to administer naloxone, and would know how to place someone who had overdosed in the recovery position. Furthermore, similar to findings from other educational interventions [11, 22], this increase in self-reported abilities aligned with a decrease in concerns about managing an opioid overdose. In particular, participants were less afraid of doing something wrong in an overdose situation and expressed fewer hesitations regarding administration of naloxone after attending the live programming. However, the level of concern did not significantly change surrounding fear of police showing up after calling emergency services, and in fact, concern about this topic was low at baseline. This is likely due to the composition of the program attendees, which majorly consisted of individuals in “helping” professions such as social workers and counselors in addition to a small percentage of law enforcement officers. Future studies can leverage and build upon these positive changes in perceived abilities and concerns amongst the helping professions to develop interdisciplinary OUD and opioid overdose prevention and response plans among local Alabama communities. Establishment of regular community-based opioid overdose simulation activities (which were not part of the OTI) may also ensure that these positive changes are translated into practical skills and maintained over time.

Despite improvements in knowledge, abilities, and concerns, and contrary to findings from previous research [22], there was no increase in participants’ readiness to intervene in an opioid overdose situation overall. This is likely because readiness was already relatively high at baseline, with 87% of participants agreeing or strongly agreeing that they could not just watch someone overdose and would have to do something to help. Of note, participants did state that they felt less likely to panic in the event of an opioid overdose after attending the program, which aligns with the decrease in overall concerns discussed above. Additionally, compared to baseline, community leaders more frequently agreed that everyone at risk of witnessing an overdose should have naloxone and that family and friends of drug users should be prepared to deal with an overdose after the conference. This suggests that the Alabama OTI was effective at engaging community leaders in a holistic approach to mitigating opioid misuse and overdose focused not only on individuals who use opioids but also on those around them who are at risk, such as children and adolescents.

Furthermore, community leaders’ intended actions related to opioid misuse and overdose mitigation after the conference exceeded their self-reported actions in the previous 6 months, with a greater proportion of attendees committed to educating caregivers about OUD and recommending naloxone when appropriate. Despite these gains, the percentage intending to take action when presented with the opportunity at least 81% of the time remained relatively low. This highlights a potential gap in infrastructure and the need for future research and community outreach. In order to bridge this gap between intended actions and remaining unmet need for action, future studies should focus on exploring, identifying, and mapping critical infrastructure and resources for opioid misuse and overdose mitigation in urban and rural Alabama communities. In fact, few studies in Alabama have investigated structural resource needs thus far [24]. Academia, researchers, and practicing healthcare professionals can serve as channels to disseminate success stories among local communities that are geographically distant and assist in adapting infrastructure and resources for implementation in local contexts, with the goal of increasing the capacity for action.

In addition to the direct benefits of the Alabama OTI on participants’ knowledge, perceptions, and intentions, our findings may improve public health on a broader scale by serving as an example for future interprofessional OUD and overdose training programs. Furthermore, the inter- and intra-professional connections made during the program have laid the foundation for sustained independent community action in mitigating opioid misuse. Future studies may leverage these connections to form community coalitions incorporating networks of support and resources for caregivers and people who use opioids across communities, particularly mental health and OUD treatment resources. Based on the findings of this study, the authors recommend two key action items and next steps for research: 1) elucidating existing opioid misuse prevention/treatment information dissemination channels among Alabama communities (e.g. public health campaigns, local organizations, state organizations, champions, peers); and 2) investigating the feasibility and acceptability of interdisciplinary community coalition formats and channels (e.g. online discussion forums, virtual working groups, live meet-and-greets). Ultimately, doing so may help to improve the scope of care for people who use opioids, the quality of life for their friends and family, and the wellbeing of individuals living in Alabama communities.

### Limitations

Several limitations are of note in this study. First, the OTI community program was limited to the state of Alabama and results may not be generalizable to other states. However, other states may find the development of the OTI’s live educational conference and web resources of interest and they can be adapted to suit the unique contexts of other regions and communities. Additionally, the authors could not control for competing education sessions or public health campaigns that OTI program attendees were exposed to during the study period. Future studies may explore the content and format of opioid-related educational programs offered in Alabama and how these programs differ or are similar to those offered in other states. Furthermore, qualitative analysis was not conducted as a follow-up to the surveys described in this article; future studies may utilize a qualitative methodology to explain or explore the quantitative survey results in more depth. Lastly, attendees’ self-reported actions and intended actions in the past/next 6 months was assessed based on the percentage of time action was/will be taken when presented with the opportunity. However, the number of times certain situations or opportunities were expected to arise based on profession was not directly measured. Future studies should investigate the anticipated frequency of OUD-related situations in different Alabama communities and by profession.

## Conclusion

The Alabama OTI improved influential community members’ knowledge, abilities, and concerns regarding management of OUD and opioid overdose. Similar programs combining live educational sessions and an interactive web-based platform can be replicated in other states. Future studies can expand upon the current findings by exploring existing and needed support structures and resources for opioid misuse prevention and treatment across diverse Alabama communities.

## Supplementary Information

**Additional file 1.** Baseline Survey. The Alabama Opioid Training Institute for Community Leaders pre-conference survey instrument.

**Additional file 2.** Post-Conference Survey. The Alabama Opioid Training Institute for Community Leaders post-conference survey instrument.

## Data Availability

The datasets generated and/or analyzed during the current study are not publicly available due to data sharing restrictions enforced by the authors’ Institutional Review Board but are available from the corresponding author on reasonable request.

## Abbreviations

CHW: Community health worker
EMT: Emergency medical technician
IRB: Institutional Review Board
MAT: Medication assisted treatment
OOAS: Opioid Overdose Attitude Scale
OOKS: Opioid Overdose Knowledge Scale
OTI: Opioid Training Institute
OUD: Opioid Use Disorder
