# Supplementary material for: A state-wide education program on opioid use disorder: influential community members’ knowledge, beliefs, and opportunities for coalition development
Source: BMC Public Health. 2022 May 4;22:886. doi: 10.1186/s12889-022-13248-z (PMC9066873; doi:10.1186/s12889-022-13248-z)
Supplement: Supplementary file 1 — Additional file 1. Baseline Survey. The Alabama Opioid Training Institute for Community Leaders pre-conference survey instrument. [file 12889_2022_13248_MOESM1_ESM.docx]

**ALABAMA OPIOID TRAINING INSTITUTE FOR COMMUNITY LEADERS**

***Baseline Survey***

1. **CONTACT INFORMATION**
2. Please provide the following information about yourself

First and Last Name, e.g. John Doe: ______________________________________________

Email address provided during registration: _________________________________________

Alabama License Number (for CE purposes): _______________________________________

1. **INFORMATION ABOUT YOU**

**For the following questions, please indicate your response by checking the appropriate box or writing your answer in the space provided.**

1. Please indicate your profession:

🞏 Alabama Coroners’ Association member 🞏 Behavioral health specialist

🞏 Civic official or city servant 🞏 Community member

🞏 Emergency Medical Technician (EMT) 🞏 Faith-based organization or church official

🞏 Guidance counselor 🞏 Law enforcement

🞏 Lawyer 🞏 Medication Assisted Therapy (MAT) provider

🞏 Mental health counselor 🞏 School coach

🞏 School nurse 🞏 School teacher (K-12)

🞏 Social worker 🞏 Other. Please specify: ______________________

1. Please indicate the place you work, e.g. school, church: __________________________________
2. Where is your place of work that you represent and/or advocate for?

_______________________________________________________________________________

*City State County*

1. Please enter your age in years: _________________
2. Please indicate your sex: 🞏 Male 🞏 Female
3. With which race do you most closely identify? Check all that apply.

🞏 White/Caucasian 🞏 Black/African American 🞏 Asian or Pacific Islander

🞏 Native American or Alaska Native 🞏 Other

1. Are you of Hispanic, Latino, or Spanish origin?: 🞏 No 🞏 Yes
2. Have you participated in any other education or training programs related to opioid misuse and abuse in the past 6 months?

🞏 No

🞏 Yes. In 1-3 sentences, please describe the topic and agency offering the training: ___________________________________________________________________________________________________________________________________________________________________________________________________________________________________________________

1. Do you know or have you ever known anyone in your personal or professional life who has struggled with opioid use disorder (OUD)? Opioid use disorder is characterized by a dependence or craving for opioid drugs that often negatively affects individuals’ social, home, school, and work life.

🞏 No

🞏 Yes

1. Do you currently offer services or programs related to opioid use disorder in your job or community?

🞏 No

🞏 Yes. 🡪 Which services? Check all that apply:

🞏 Methadone program or provision

🞏 Buprenorphine or buprenorphine/naloxone provision

🞏 Needle exchange program or sell syringes

🞏 Cognitive behavioral therapy or counseling

🞏 Medication disposal or drug take-back service

🞏 Education sessions or programs

🞏 Other. Please specify: _______________________________________

1. **WHAT YOU KNOW ABOUT OPIOID-RELATED ISSUES**
2. Fentanyl is the number one drug leading to opioid overdose deaths nationwide.

🞏 True 🞏 False

1. Multiple doses of naloxone may not be effective in reversing overdose from the following opioid:

🞏 Fentanyl 🞏 Carfentanil 🞏 Heroin 🞏 Oxycodone

1. Which of the following mental and social factors are shown to influence risk for opioid misuse, especially in adolescents?

🞏 Level of self-esteem 🞏 Resiliency (coping and problem-solving skills)

🞏 Stress or feelings of inadequacy 🞏 Behavioral disorders

🞏 Bullying

🞏 All of the above

1. Over time, opioid use disorder affects individuals’ ability to:

🞏 Regulate behavior 🞏 Make decisions 🞏 Respond to stressful situations

🞏 All of the above

1. Which of the following are indicators of an opioid overdose? Check all that apply.

🞏 Having blood-shot eyes 🞏 Slow or shallow breathing 🞏 Lips, hands or feet turning blue

🞏 Loss of consciousness 🞏 Unresponsive 🞏 Seizing

🞏 Deep snoring 🞏 Very small pupils 🞏 Agitated behavior

🞏 Rapid heartbeat

1. Which of the following should be done when managing a heroin / opioid overdose? Check all that apply.

🞏 Call an ambulance (911) 🞏 Inject the person with salt solution or milk

🞏 Give stimulants (e.g. cocaine or black coffee) 🞏 Give naloxone (opioid overdose antidote)

🞏 Put the person in a bath of cold water 🞏 Put the person in bed to sleep it off

🞏 Stay with the person until an ambulance arrives

🞏 Check for responsiveness (yell their name, rub the center of the chest)

🞏 Give chest compressions and/or rescue breathing if the person is not breathing (CPR)

1. What is naloxone used for?

🞏 To reverse the effects of an opioid overdose (e.g. heroin, methadone)

🞏 To reverse the effects of an amphetamine overdose

🞏 To reverse the effects of a cocaine overdose

🞏 To reverse the effects of any overdose

1. How long does naloxone take to have an effect?

🞏 Within 5 minutes 🞏 6-10 minutes 🞏 11-20 minutes 🞏 21-40 minutes

1. How long do the effects of naloxone last for?

🞏 Less than 20 minutes 🞏 30-90 minutes 🞏 2 to 6 hours 🞏 6 to 12 hours

1. Which of the following is NOT used in medication assisted therapy (MAT) to treat opioid use disorder?

🞏 Hydromorphone 🞏 Buprenorphine-containing products 🞏 Methadone 🞏 Naltrexone

1. Methadone is the treatment of choice for pregnant women with opioid use disorder.

🞏 True 🞏 False

1. Which of the following is a 12-step program developed to help individuals with substance use disorder?

🞏 Narcotics Anonymous 🞏 Motivational Interviewing

🞏 Mindfulness Meditation 🞏 Dialectical Behavioral Therapy

1. Some individuals may use more opioids in an attempt to relieve depression that occurs with their chronic pain.

🞏 True 🞏 False

1. **YOUR ACTIONS IN THE PAST 6 MONTHS**

**Instructions:** Thinking of opportunities you may have had in the past 6 months, please rate how frequently or infrequently you provided the following opioid-related services when the opportunity presented itself from 0-20%, 21-40%, 41-60%, 61-80%, or 81-100% of the time.

|  | 0-20% of the time | 21-40% of the time | 41-60% of the time | 61-80% of the time | 81-100% of the time | No Opportunities / Not Applicable |
| --- | --- | --- | --- | --- | --- | --- |
| Screened or assessed someone for potential opioid use disorder (OUD) or opioid overdose risk | 🞏 | 🞏 | 🞏 | 🞏 | 🞏 | 🞏 |
| Educated people about OUD through school or community-based programs | 🞏 | 🞏 | 🞏 | 🞏 | 🞏 | 🞏 |
| Provided education or counseling to family or caregivers regarding OUD | 🞏 | 🞏 | 🞏 | 🞏 | 🞏 | 🞏 |
| Recommended or discussed specialized treatment or rehabilitation facilities for a person with OUD | 🞏 | 🞏 | 🞏 | 🞏 | 🞏 | 🞏 |
| Recommended or discussed cognitive behavioral therapy for OUD | 🞏 | 🞏 | 🞏 | 🞏 | 🞏 | 🞏 |
| Recommended or discussed medication assisted treatment for OUD | 🞏 | 🞏 | 🞏 | 🞏 | 🞏 | 🞏 |
| Recommended or discussed naloxone | 🞏 | 🞏 | 🞏 | 🞏 | 🞏 | 🞏 |
| Spoke with a healthcare provider on someone’s behalf | 🞏 | 🞏 | 🞏 | 🞏 | 🞏 | 🞏 |

1. **YOUR ABILITIES, BELIEFS, AND READINESS**

**Instructions:** On a scale of 1 to 7, please rate your level of agreement or disagreement with the following statements, with 1 being strongly disagree to 7 being strongly agree.

| ***Ability to Manage an Opioid Overdose:*** | Strongly Disagree | Disagree | Somewhat Disagree | Neutral | Somewhat Agree | Agree | Strongly Agree |
| --- | --- | --- | --- | --- | --- | --- | --- |
| I already have enough information about how to manage an overdose | 🞏 | 🞏 | 🞏 | 🞏 | 🞏 | 🞏 | 🞏 |
| I am already able to administer naloxone to someone who has overdosed | 🞏 | 🞏 | 🞏 | 🞏 | 🞏 | 🞏 | 🞏 |
| I would be able to check that someone who has overdosed was breathing properly | 🞏 | 🞏 | 🞏 | 🞏 | 🞏 | 🞏 | 🞏 |
| I am going to need more training before I would feel confident to help someone who has overdosed | 🞏 | 🞏 | 🞏 | 🞏 | 🞏 | 🞏 | 🞏 |
| I would be able to perform mouth-to-mouth resuscitation on someone who has overdosed | 🞏 | 🞏 | 🞏 | 🞏 | 🞏 | 🞏 | 🞏 |
| I would be able to perform chest compressions on someone who has overdosed | 🞏 | 🞏 | 🞏 | 🞏 | 🞏 | 🞏 | 🞏 |
| If someone overdoses, I would know what to do to help them | 🞏 | 🞏 | 🞏 | 🞏 | 🞏 | 🞏 | 🞏 |
| I would be able to place someone who has overdosed in the recovery position | 🞏 | 🞏 | 🞏 | 🞏 | 🞏 | 🞏 | 🞏 |
| I know very little about how to help someone who has overdosed | 🞏 | 🞏 | 🞏 | 🞏 | 🞏 | 🞏 | 🞏 |
| I would be able to deal effectively with an overdose | 🞏 | 🞏 | 🞏 | 🞏 | 🞏 | 🞏 | 🞏 |

| ***Beliefs About Managing an Opioid Overdose:*** | Strongly Disagree | Disagree | Somewhat Disagree | Neutral | Somewhat Agree | Agree | Strongly Agree |
| --- | --- | --- | --- | --- | --- | --- | --- |
| I would be afraid of giving naloxone in case the person becomes aggressive afterwards | 🞏 | 🞏 | 🞏 | 🞏 | 🞏 | 🞏 | 🞏 |
| I would be afraid of doing something wrong in an overdose situation | 🞏 | 🞏 | 🞏 | 🞏 | 🞏 | 🞏 | 🞏 |
| I would be reluctant to use naloxone for fear of precipitating withdrawal symptoms | 🞏 | 🞏 | 🞏 | 🞏 | 🞏 | 🞏 | 🞏 |
| I would be concerned about calling emergency services in case the police show up | 🞏 | 🞏 | 🞏 | 🞏 | 🞏 | 🞏 | 🞏 |
| If I tried to help someone who has overdosed, I might accidentally hurt them | 🞏 | 🞏 | 🞏 | 🞏 | 🞏 | 🞏 | 🞏 |
| I would feel safer if I knew that naloxone was around | 🞏 | 🞏 | 🞏 | 🞏 | 🞏 | 🞏 | 🞏 |
| I would be afraid of suffering a needle stick injury if I had to give someone a naloxone injection | 🞏 | 🞏 | 🞏 | 🞏 | 🞏 | 🞏 | 🞏 |
| Needles frighten me, and I wouldn’t be able to give someone an injection of naloxone | 🞏 | 🞏 | 🞏 | 🞏 | 🞏 | 🞏 | 🞏 |

| ***Readiness to Intervene in an Opioid Overdose:*** | Strongly Disagree | Disagree | Somewhat Disagree | Neutral | Somewhat Agree | Agree | Strongly Agree |
| --- | --- | --- | --- | --- | --- | --- | --- |
| Everyone at risk of witnessing an overdose should have naloxone | 🞏 | 🞏 | 🞏 | 🞏 | 🞏 | 🞏 | 🞏 |
| I couldn’t just watch someone overdose, I would have to do something to help | 🞏 | 🞏 | 🞏 | 🞏 | 🞏 | 🞏 | 🞏 |
| If someone overdoses, I would call an ambulance, but I wouldn’t be willing to do anything else | 🞏 | 🞏 | 🞏 | 🞏 | 🞏 | 🞏 | 🞏 |
| Family and friends of drug users should be prepared to deal with an overdose | 🞏 | 🞏 | 🞏 | 🞏 | 🞏 | 🞏 | 🞏 |
| If I saw an overdose, I would panic and not be able to help | 🞏 | 🞏 | 🞏 | 🞏 | 🞏 | 🞏 | 🞏 |
| If I witnessed an overdose, I would call an ambulance immediately | 🞏 | 🞏 | 🞏 | 🞏 | 🞏 | 🞏 | 🞏 |
| I would stay with the overdose victim until help arrives | 🞏 | 🞏 | 🞏 | 🞏 | 🞏 | 🞏 | 🞏 |
| If I saw an overdose, I would feel nervous, but I would still take the necessary actions | 🞏 | 🞏 | 🞏 | 🞏 | 🞏 | 🞏 | 🞏 |
| I will do whatever is necessary to save someone’s life in an overdose situation | 🞏 | 🞏 | 🞏 | 🞏 | 🞏 | 🞏 | 🞏 |
| If someone overdoses, I want to be able to help them | 🞏 | 🞏 | 🞏 | 🞏 | 🞏 | 🞏 | 🞏 |
