# Supplementary material for: A state-wide education program on opioid use disorder: influential community members’ knowledge, beliefs, and opportunities for coalition development
Source: BMC Public Health. 2022 May 4;22:886. doi: 10.1186/s12889-022-13248-z (PMC9066873; doi:10.1186/s12889-022-13248-z)
Supplement: Supplementary file 2 — Additional file 2. Post-Conference Survey. The Alabama Opioid Training Institute for Community Leaders post-conference survey instrument. [file 12889_2022_13248_MOESM2_ESM.docx]

**ALABAMA OPIOID TRAINING INSTITUTE FOR COMMUNITY LEADERS**

***Post-Conference Survey***

1. **CONTACT INFORMATION**
2. Please provide the following information about yourself

First and Last Name, e.g. John Doe: ______________________________________________

Email address provided during registration:_________________________________________

Alabama License Number (for CE purposes): _______________________________________

1. **WHAT YOU KNOW ABOUT OPIOID-RELATED ISSUES**
2. Fentanyl is the number one drug leading to opioid overdose deaths nationwide.

🞏 True 🞏 False

1. Multiple doses of naloxone may not be effective in reversing overdose from the following opioid:

🞏 Fentanyl 🞏 Carfentanil 🞏 Heroin 🞏 Oxycodone

1. Which of the following mental and social factors are shown to influence risk for opioid misuse, especially in adolescents?

🞏 Level of self-esteem 🞏 Resiliency (coping and problem-solving skills)

🞏 Stress or feelings of inadequacy 🞏 Behavioral disorders

🞏 Bullying

🞏 All of the above

1. Over time, opioid use disorder affects individuals’ ability to:

🞏 Regulate behavior 🞏 Make decisions 🞏 Respond to stressful situations

🞏 All of the above

1. Which of the following are indicators of an opioid overdose? Check all that apply.

🞏 Having blood-shot eyes 🞏 Slow or shallow breathing 🞏 Lips, hands or feet turning blue

🞏 Loss of consciousness 🞏 Unresponsive 🞏 Seizing

🞏 Deep snoring 🞏 Very small pupils 🞏 Agitated behavior

🞏 Rapid heartbeat

1. Which of the following should be done when managing a heroin / opioid overdose? Check all that apply.

🞏 Call an ambulance (911) 🞏 Inject the person with salt solution or milk

🞏 Give stimulants (e.g. cocaine or black coffee) 🞏 Give naloxone (opioid overdose antidote)

🞏 Put the person in a bath of cold water 🞏 Put the person in bed to sleep it off

🞏 Stay with the person until an ambulance arrives

🞏 Check for responsiveness (yell their name, rub the center of the chest)

🞏 Give chest compressions and/or rescue breathing if the person is not breathing (CPR)

1. What is naloxone used for?

🞏 To reverse the effects of an opioid overdose (e.g. heroin, methadone)

🞏 To reverse the effects of an amphetamine overdose

🞏 To reverse the effects of a cocaine overdose

🞏 To reverse the effects of any overdose

1. How long does naloxone take to have an effect?

🞏 Within 5 minutes 🞏 6-10 minutes 🞏 11-20 minutes 🞏 21-40 minutes

1. How long do the effects of naloxone last for?

🞏 Less than 20 minutes 🞏 30-90 minutes 🞏 2 to 6 hours 🞏 6 to 12 hours

1. Which of the following is NOT used in medication assisted therapy (MAT) to treat opioid use disorder?

🞏 Hydromorphone 🞏 Buprenorphine-containing products 🞏 Methadone 🞏 Naltrexone

1. Methadone is the treatment of choice for pregnant women with opioid use disorder.

🞏 True 🞏 False

1. Which of the following is a 12-step program developed to help individuals with substance use disorder?

🞏 Narcotics Anonymous 🞏 Motivational Interviewing

🞏 Mindfulness Meditation 🞏 Dialectical Behavioral Therapy

1. Some individuals may use more opioids in an attempt to relieve depression that occurs with their chronic pain.

🞏 True 🞏 False

1. **YOUR INTENDED ACTIONS IN THE NEXT 6 MONTHS**

**Instructions:** Thinking of opportunities you may have in the next 6 months, please rate how frequently or infrequently you intend to provide the following opioid-related services when the opportunity presents itself from 0-20%, 21-40%, 41-60%, 61-80%, or 81-100% of the time.

|  | 0-20% of the time | 21-40% of the time | 41-60% of the time | 61-80% of the time | 81-100% of the time | No Opportunities / Not Applicable |
| --- | --- | --- | --- | --- | --- | --- |
| Screen or assess someone for potential opioid use disorder (OUD) or opioid overdose risk | 🞏 | 🞏 | 🞏 | 🞏 | 🞏 | 🞏 |
| Educate people about OUD through school or community-based programs | 🞏 | 🞏 | 🞏 | 🞏 | 🞏 | 🞏 |
| Provide education or counseling to family or caregivers regarding OUD | 🞏 | 🞏 | 🞏 | 🞏 | 🞏 | 🞏 |
| Recommend or discuss specialized treatment or rehabilitation facilities for a person with OUD | 🞏 | 🞏 | 🞏 | 🞏 | 🞏 | 🞏 |
| Recommend or discuss cognitive behavioral therapy for OUD | 🞏 | 🞏 | 🞏 | 🞏 | 🞏 | 🞏 |
| Recommend or discuss medication assisted treatment for OUD | 🞏 | 🞏 | 🞏 | 🞏 | 🞏 | 🞏 |
| Recommend or discuss naloxone | 🞏 | 🞏 | 🞏 | 🞏 | 🞏 | 🞏 |
| Speak with a healthcare provider on someone’s behalf | 🞏 | 🞏 | 🞏 | 🞏 | 🞏 | 🞏 |

1. **YOUR ABILITIES, BELIEFS, AND READINESS**

**Instructions:** On a scale of 1 to 7, please rate your level of agreement or disagreement with the following statements, with 1 being strongly disagree to 7 being strongly agree.

| ***Ability to Manage an Opioid Overdose:*** | Strongly Disagree | Disagree | Somewhat Disagree | Neutral | Somewhat Agree | Agree | Strongly Agree |
| --- | --- | --- | --- | --- | --- | --- | --- |
| I already have enough information about how to manage an overdose | 🞏 | 🞏 | 🞏 | 🞏 | 🞏 | 🞏 | 🞏 |
| I am already able to administer naloxone to someone who has overdosed | 🞏 | 🞏 | 🞏 | 🞏 | 🞏 | 🞏 | 🞏 |
| I would be able to check that someone who has overdosed was breathing properly | 🞏 | 🞏 | 🞏 | 🞏 | 🞏 | 🞏 | 🞏 |
| I am going to need more training before I would feel confident to help someone who has overdosed | 🞏 | 🞏 | 🞏 | 🞏 | 🞏 | 🞏 | 🞏 |
| I would be able to perform mouth-to-mouth resuscitation on someone who has overdosed | 🞏 | 🞏 | 🞏 | 🞏 | 🞏 | 🞏 | 🞏 |
| I would be able to perform chest compressions on someone who has overdosed | 🞏 | 🞏 | 🞏 | 🞏 | 🞏 | 🞏 | 🞏 |
| If someone overdoses, I would know what to do to help them | 🞏 | 🞏 | 🞏 | 🞏 | 🞏 | 🞏 | 🞏 |
| I would be able to place someone who has overdosed in the recovery position | 🞏 | 🞏 | 🞏 | 🞏 | 🞏 | 🞏 | 🞏 |
| I know very little about how to help someone who has overdosed | 🞏 | 🞏 | 🞏 | 🞏 | 🞏 | 🞏 | 🞏 |
| I would be able to deal effectively with an overdose | 🞏 | 🞏 | 🞏 | 🞏 | 🞏 | 🞏 | 🞏 |

| ***Beliefs About Managing an Opioid Overdose:*** | Strongly Disagree | Disagree | Somewhat Disagree | Neutral | Somewhat Agree | Agree | Strongly Agree |
| --- | --- | --- | --- | --- | --- | --- | --- |
| I would be afraid of giving naloxone in case the person becomes aggressive afterwards | 🞏 | 🞏 | 🞏 | 🞏 | 🞏 | 🞏 | 🞏 |
| I would be afraid of doing something wrong in an overdose situation | 🞏 | 🞏 | 🞏 | 🞏 | 🞏 | 🞏 | 🞏 |
| I would be reluctant to use naloxone for fear of precipitating withdrawal symptoms | 🞏 | 🞏 | 🞏 | 🞏 | 🞏 | 🞏 | 🞏 |
| I would be concerned about calling emergency services in case the police show up | 🞏 | 🞏 | 🞏 | 🞏 | 🞏 | 🞏 | 🞏 |
| If I tried to help someone who has overdosed, I might accidentally hurt them | 🞏 | 🞏 | 🞏 | 🞏 | 🞏 | 🞏 | 🞏 |
| I would feel safer if I knew that naloxone was around | 🞏 | 🞏 | 🞏 | 🞏 | 🞏 | 🞏 | 🞏 |
| I would be afraid of suffering a needle stick injury if I had to give someone a naloxone injection | 🞏 | 🞏 | 🞏 | 🞏 | 🞏 | 🞏 | 🞏 |
| Needles frighten me, and I wouldn’t be able to give someone an injection of naloxone | 🞏 | 🞏 | 🞏 | 🞏 | 🞏 | 🞏 | 🞏 |

| ***Readiness to Intervene in an Opioid Overdose:*** | Strongly Disagree | Disagree | Somewhat Disagree | Neutral | Somewhat Agree | Agree | Strongly Agree |
| --- | --- | --- | --- | --- | --- | --- | --- |
| Everyone at risk of witnessing an overdose should have naloxone | 🞏 | 🞏 | 🞏 | 🞏 | 🞏 | 🞏 | 🞏 |
| I couldn’t just watch someone overdose, I would have to do something to help | 🞏 | 🞏 | 🞏 | 🞏 | 🞏 | 🞏 | 🞏 |
| If someone overdoses, I would call an ambulance, but I wouldn’t be willing to do anything else | 🞏 | 🞏 | 🞏 | 🞏 | 🞏 | 🞏 | 🞏 |
| Family and friends of drug users should be prepared to deal with an overdose | 🞏 | 🞏 | 🞏 | 🞏 | 🞏 | 🞏 | 🞏 |
| If I saw an overdose, I would panic and not be able to help | 🞏 | 🞏 | 🞏 | 🞏 | 🞏 | 🞏 | 🞏 |
| If I witnessed an overdose, I would call an ambulance immediately | 🞏 | 🞏 | 🞏 | 🞏 | 🞏 | 🞏 | 🞏 |
| I would stay with the overdose victim until help arrives | 🞏 | 🞏 | 🞏 | 🞏 | 🞏 | 🞏 | 🞏 |
| If I saw an overdose, I would feel nervous, but I would still take the necessary actions | 🞏 | 🞏 | 🞏 | 🞏 | 🞏 | 🞏 | 🞏 |
| I will do whatever is necessary to save someone’s life in an overdose situation | 🞏 | 🞏 | 🞏 | 🞏 | 🞏 | 🞏 | 🞏 |
| If someone overdoses, I want to be able to help them | 🞏 | 🞏 | 🞏 | 🞏 | 🞏 | 🞏 | 🞏 |

1. **SATISFACTION WITH THE PROGRAM**

**Instructions:** On a scale of 1 to 7, please rate your level of agreement or disagreement with the following statements regarding the educational program, with 1 being strongly disagree and 7 being strongly agree.

|  | Strongly Disagree | Disagree | Somewhat Disagree | Neutral | Somewhat Agree | Agree | Strongly Agree |
| --- | --- | --- | --- | --- | --- | --- | --- |
| All learning objectives for this educational program were met | 🞏 | 🞏 | 🞏 | 🞏 | 🞏 | 🞏 | 🞏 |
| Content was relevant to my job | 🞏 | 🞏 | 🞏 | 🞏 | 🞏 | 🞏 | 🞏 |
| Realistic time was allowed for the training | 🞏 | 🞏 | 🞏 | 🞏 | 🞏 | 🞏 | 🞏 |
| The training materials were easy to read | 🞏 | 🞏 | 🞏 | 🞏 | 🞏 | 🞏 | 🞏 |
| The training adequately described strategies to prevent opioid use disorder (OUD) | 🞏 | 🞏 | 🞏 | 🞏 | 🞏 | 🞏 | 🞏 |
| The training adequately described strategies to treat OUD | 🞏 | 🞏 | 🞏 | 🞏 | 🞏 | 🞏 | 🞏 |
| The training adequately described strategies to communicate with individuals with OUD | 🞏 | 🞏 | 🞏 | 🞏 | 🞏 | 🞏 | 🞏 |
| After the training, my ability to recommend resources to individuals with OUD increased | 🞏 | 🞏 | 🞏 | 🞏 | 🞏 | 🞏 | 🞏 |
| After the training, my ability to recommend treatment to individuals with OUD increased | 🞏 | 🞏 | 🞏 | 🞏 | 🞏 | 🞏 | 🞏 |
| After the training, my ability to collaborate with others to prevent OUD increased | 🞏 | 🞏 | 🞏 | 🞏 | 🞏 | 🞏 | 🞏 |

1. **GENERAL QUESTIONS ABOUT THE PROGRAM**
2. **Instructions:** On a scale of 1 to 7, please rate your level of agreement or disagreement with the following statements regarding the educational program, with 1 being strongly disagree and 7 being strongly agree.

|  | Strongly Disagree | Disagree | Somewhat Disagree | Neutral | Somewhat Agree | Agree | Strongly Agree |
| --- | --- | --- | --- | --- | --- | --- | --- |
| The training content was clear and concise | 🞏 | 🞏 | 🞏 | 🞏 | 🞏 | 🞏 | 🞏 |
| Realistic time was allowed for the training | 🞏 | 🞏 | 🞏 | 🞏 | 🞏 | 🞏 | 🞏 |
| I was satisfied with the material presented during the program | 🞏 | 🞏 | 🞏 | 🞏 | 🞏 | 🞏 | 🞏 |
| I would recommend this program to others | 🞏 | 🞏 | 🞏 | 🞏 | 🞏 | 🞏 | 🞏 |
| The training met my educational needs | 🞏 | 🞏 | 🞏 | 🞏 | 🞏 | 🞏 | 🞏 |
| The quality of the facility was excellent | 🞏 | 🞏 | 🞏 | 🞏 | 🞏 | 🞏 | 🞏 |
| I have been pleased with the communication regarding the program | 🞏 | 🞏 | 🞏 | 🞏 | 🞏 | 🞏 | 🞏 |
| I have been pleased with the registration process for the program | 🞏 | 🞏 | 🞏 | 🞏 | 🞏 | 🞏 | 🞏 |
| The presenters were engaging | 🞏 | 🞏 | 🞏 | 🞏 | 🞏 | 🞏 | 🞏 |

1. How did you hear about this program?: _________________________________________________
2. Please provide any comments you have about the program:
3. Please enter the **ATTENDANCE CODE** provided at the end of the program: _______________
